# An audit looking at the impact of poverty on referrals to child and adolescent mental health services

**DOI:** 10.1192/bjo.2021.886

**Published:** 2021-06-18

**Authors:** Aida Nourbakhsh, Kandarp Joshi, Breige Yorston

**Affiliations:** 1 University of Aberdeen; 2 Child and Adolescent Mental Health Services NHS Grampian, University of Aberdeen; 3 CAMHS NHS Grampian

## Abstract

**Aims:**

Recently, there has been a greater focus on how mental health in young people (YP) can be improved. Up to 10% of YP in Scotland have a diagnosable mental health condition1 and half of all adults with mental ill-health have had symptoms from their mid-teens2. Poverty is an important factor associated with poorer mental well-being from an early age which worsens if left untreated3. The aim of this audit was to answer the question: Are more YP referred from the least deprived areas, and are they more likely to require medication intervention or high intensity (tier 4) care? The results of which could help identify possible avenues for intervention to help improve retention of those most at risk of negative outcomes.

**Method:**

NHS Grampian CAMHS provides service to Aberdeen City, Aberdeenshire, and Moray. Pre-collected data over 15 months from these areas were analysed using the Scottish Index of Multiple Deprivation (SIMD) deciles to distinguish any differences between referrals made. In addition, this audit evaluated the data to define any trends of deprivation linking YP to medication intervention or tier 4 care.

**Result:**

Results showed that more referrals were made for YP in low-ranking areas (3.19% of decile one compared to 1.74% of decile ten). The referrals were also more likely to be rejected based on the referral criteria, 33% in decile one versus 21% in decile ten. The increased rejection of referrals is most likely a reflection of the health inequalities faced by communities in more deprived areas. In terms of service provision, the patients from the most deprived areas are 3 times more like to require tier 4 care while the least deprived are 1.5 times more likely as compared to percentage of population. With regards to medication intervention patients from deciles one, five, six and seven have significantly higher numbers.

**Conclusion:**

This project set out to look at the current service provided by CAMHS and found that despite best efforts deprivation has had an impact on the acceptance of referrals. Going forward this data will be shared with multiagency stakeholders to develop service provisions, in particular the issues identified with the rejection of referrals in more deprived areas. Higher level of medication use in more deprived population is not unexpected but highlights the need to share the findings with a multiagency network.

